# A Novel Multiobjective Evolutionary Algorithm Based on Regression Analysis

**DOI:** 10.1155/2015/439307

**Published:** 2015-03-22

**Authors:** Zhiming Song, Maocai Wang, Guangming Dai, Massimiliano Vasile

**Affiliations:** ^1^School of Computer, China University of Geosciences, Wuhan 430074, China; ^2^Department of Mechanical & Aerospace Engineering, University of Strathclyde, Glasgow G1 1XJ, UK

## Abstract

As is known, the Pareto set of a continuous multiobjective optimization problem with *m* objective functions is a piecewise continuous (*m* − 1)-dimensional manifold in the decision space under some mild conditions. However, how to utilize the regularity to design multiobjective optimization algorithms has become the research focus. In this paper, based on this regularity, a model-based multiobjective evolutionary algorithm with regression analysis (MMEA-RA) is put forward to solve continuous multiobjective optimization problems with variable linkages. In the algorithm, the optimization problem is modelled as a promising area in the decision space by a probability distribution, and the centroid of the probability distribution is (*m* − 1)-dimensional piecewise continuous manifold. The least squares method is used to construct such a model. A selection strategy based on the nondominated sorting is used to choose the individuals to the next generation. The new algorithm is tested and compared with NSGA-II and RM-MEDA. The result shows that MMEA-RA outperforms RM-MEDA and NSGA-II on the test instances with variable linkages. At the same time, MMEA-RA has higher efficiency than the other two algorithms. A few shortcomings of MMEA-RA have also been identified and discussed in this paper.

## 1. Introduction

Evolutionary algorithm has become an increasingly popular design and optimization tool in the last few years [1]. Although there have been a lot of researches about evolutionary algorithm, there are still many new areas that needed to be explored with sufficient depth. One of them is how to use the evolutionary algorithm to solve multiobjective optimization problems. The first implementation of a multiobjective evolutionary algorithm dates back to the mid-1980s [2]. Since then, many researchers have done a considerable amount of works in the area, which is known as multiobjective evolutionary algorithm (MOEA).

Because of the ability to deal with a set of possible solutions simultaneously, evolutionary algorithm seems particularly suitable to solve multiobjective optimization problems. The ability makes it possible to search several members of the Pareto-optimal set in a single run of the algorithm [3]. Obviously, evolutionary algorithm is more effective than the traditional mathematical programming methods in solving multiobjective optimization problem because the traditional methods need to perform a series of separate runs [4].

The current MOEA research mainly focuses on some highly related issues [5]. The first issue is the fitness assignment and diversity maintenance. Some techniques such as fitness sharing and crowding have been frequently used to maintain the diversity of the search. The second issue is the external population. The external population is used to record nondominated solutions found during the search. There have been some efforts on how to maintain and utilize such an external population. The last issue is the combination of MOEA and local search. Researches have shown that the combination of evolutionary algorithm and local heuristics search outperforms traditional evolutionary algorithms in a wide variety of scalar objective optimization problems [4, 6].

However, there are little researches focusing on the way to generate new solutions in MOEA. Currently, most MOEAs directly adopt traditional genetic operators such as crossover and mutation. These methods have not fully utilized the characteristics of MOP when generating new solutions. Some researches show that MOEA fails to solve MOPs with variable linkages, and the recombination operators are crucial to the performance of MOEA [7]. It has been noted that under mild smoothness conditions, the Pareto set (PS) of a continuous MOP is a piecewise continuous (*m* − 1)-dimensional manifold, where *m* is the number of the objectives. However, as analyzed in [8], this regularity has not been exploited explicitly by most current MOEA.

In 2005, Zhou et al. proposed to extract regularity patterns of the Pareto set by using local principal component analysis (PCA) [9]. They had also studied two naive hybrid MOEAs. In the two MOEAs, some trial solutions were generated by traditional genetic operators and others by sampling from probability models based on regularity patterns in 2006 [10].

In 2007, Zhang et al. conducted a further and thorough investigation along their previous works in [9, 10]. They proposed a regularity model-based multiobjective estimation of distribution algorithm and named it as RM-MEDA [5]. At each generation, the proposed algorithm models a promising area in the decision space by a probability distribution whose centroid is a (*m* − 1)-dimensional piecewise continuous manifold. The local principal component analysis algorithm is used to build such a model. Systematic experiments have shown that RM-MEDA outperforms some other algorithms on a set of test instances with variable linkages.

In 2008, Zhou et al. proposed a probabilistic model based multiobjective evolutionary algorithm to approximate PS and PF (Pareto front) for a MOP in this class simultaneously and named the algorithm as MMEA [11]. They proposed two typical classes of continuous MOPs as follows. One class is that PS and PF are of the same dimensionality while the other one is that PF is a (*m* − 1)-dimensional continuous manifold and PS is a continuous manifold with a higher dimensionality. There is a class of MOPs, in which the dimensionalities of PS and PF are different so that a good approximation to PF might not approximate PS very well. MMEA could promote the population diversity both in the decision spaces and in the objective spaces.

Modeling method is a crucial part for MOEA because it determines the performance of the algorithms. Zhang et al. built such a model by local principal component analysis (PCA) algorithm [5]. The test results show that the method has great performance over some instances with linkage variables. However, there are still some shortcomings about the method. The first shortcoming is that RM-MEDA needs extra CPU time for running local PCA at each generation. The second one is that the model is just linear fitting for all types of PS, including the one with nonlinear linkage variables, which enable that the result may be not accurate.

In the paper, we proposed a model-based multiobjective evolutionary algorithm with regression analysis, which is named as MMEA-RA. In MMEA-RA, a new modeling method based on regression analysis is put forward. In the method, least squares method (LSM) is used to fit a 1-dimensional manifold in high-dimensional space. Because least squares can fit any type of curves through its model, the shortcomings of RM-MEDA can be avoided, especially for the instances with nonlinkage variables.

The rest of this paper is organized as follows. After defining the continuous multiobjective optimization problem in Section 2, the new model of multiobjective evolutionary algorithm based on regression analysis is put forward in Section 3. Then, a description of the test cases for MMEA-RA follows in Section 4. After presenting the results of the tests, the performance of MMEA-RA is analyzed and some conclusions are given in Section 5.

## 2. Problem Definition

In this paper, the continuous multiobjective optimization problem is defined as follows [5]:

(1) min⁡ Fx=f1x,f2x,…,fmxT s.t. x=x1,…,xnT∈X,

where *X* ⊂ *R* ^*n*^ is the decision space and *x* = (*x*_1_,…, *x*_*n*_)^*T*^ is the decision vector. *F* : *X* → *R* ^*m*^ consists of *m* real-valued continuous objective functions *f*_*i*_(*x*)  (*i* = 1,…, *m*). *R* ^*m*^ is the objective space.

Let *a* = (*a*_1_,…, *a*_*n*_)^*T*^ ∈ *R* ^*n*^ and *b* = (*b*_1_,…, *b*_*n*_)^*T*^ ∈ *R* ^*n*^ be two vectors, and *a* is said to dominate *b*, denoted by *a*≺*b*, if *a*_*i*_ ≤ *b*_*i*_ for all *i* = 1,…, *n*, and *a* ≠ *b*. A point *x* ^*^ ∈ *X* is called (globally) Pareto optimal if there is no *x* ∈ *X* such that *F*(*x*)≺*F*(*x* ^*^). The set of all Pareto-optimal points, denoted by PS, is called the Pareto set. The set of all Pareto objective vectors is called the Pareto front, denoted by PF.

## 3. Algorithm

### 3.1. Basic Idea

Under certain smoothness assumptions, it can be induced from the Karush-Kuhn-Tucker condition that the PS of a continuous MOP defines a piecewise continuous (*m* − 1)-dimensional manifold in the decision space [12]. Therefore, the PS of a continuous biobjective optimization problem is a piecewise continuous curve in *R* ^2^.

**Figure 1:**
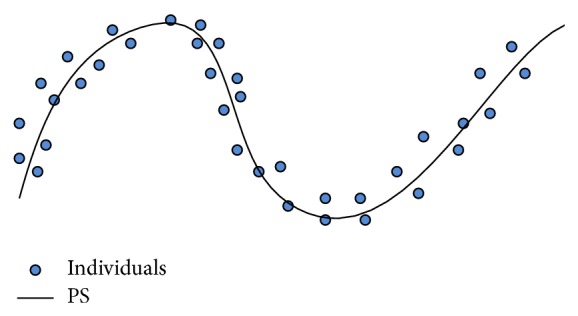
Individual solutions should be scattered around the PS in the decision space in a successful MOEA.

The population in the decision space in a MOEA for (1) will hopefully approximate the PS and is uniformly scattered around the PS as the search goes on. Therefore, we can envisage the points in the population as independent observations of a random vector *ξ* ∈ *R* ^*n*^ whose centroid is the PS of (1). Since the PS is a (*m* − 1)-dimensional piecewise continuous manifold, *ξ* can be naturally described by

(2) $$\begin{array}{c} \xi =\zeta +\varepsilon , \end{array}$$

where *ζ* is uniformly distributed over a piecewise continuous (*m* − 1)-dimensional manifold, and *ε* is an *n*-dimensional zero-mean noise vector.  Figure 1 illustrates the basic idea.

### 3.2. Algorithm Framework

In this paper, a model-based multiobjective evolutionary algorithm based on regression analysis is put forward to solve continuous multiobjective optimization problems with variable linkages. The algorithm is named as MMEA-RA. The algorithm works as follows.

*MMEA-RA*

**Step 1 (initializing).**

Set *t* = 0. Generate an initial population Pop(0) and compute the value *F* of each individual solution in Pop(0).

**Step 2 (stopping).**

If stopping condition is met, the algorithm stops and returns the nondominated solutions in Pop(*t*), and their corresponding *F* vectors constitute an approximation to the PF.

**Step 3 (modeling).**

Build the probability model in Pop(*t*) to fit expression (2),

(3.1) to compute the coefficients *a*_*k*_ for *k* = 0,1,…, *j* by solving the matrix in expression (10);
(3.2) to compute the manifold *ψ* = {*x* = (*x*_1_,…, *x*_*n*_) ∈ *R* ^*n*^} by expression (11);
(3.3) to generate a *n*-dimensional zero-mean noise vector between (−noise, noise) randomly based on expressions (13) and (14).

**Step 4 (reproducing).**

Generate a new solution set *Q* from expression (2). Evaluate the value *F* of each solution in *Q*.

**Step 5 (selecting).**

Select *N* individuals from *Q* ∪ Pop(*t*) to create Pop(*t* + 1).

**Step 6 .**

Set *t* = *t* + 1 and go to Step 2.

In the following Section 3.3, the implementation of modeling, reproducing, and selecting of the above algorithm will be given in detail.

### 3.3. Modeling

Fitting expression (2) to the points in Pop(*t*) is highly related to principal curve analysis, which aims at finding a central curve of a set of points in *R* ^*n*^ [13]. However, most current algorithms for principal curve analysis are rather expensive due to the intrinsic complexity of their models. RM-MEDA uses the (*m* − 1)-dimensional local principal component analysis (PCA) algorithm [14]; it is less complex compared with most algorithms for principal curve analysis. However, it needs much more CPU time compared with the traditional evolutionary algorithms which adopt genetic recombination operators such as crossover and mutation. Moreover, the PS cannot be exactly described by local PCA because it only uses linear curves to approximate the model at one cluster of Pop(*t*).

In this implementation, we do not make use of clustering method in the modeling process. We try to find the principal curve of the whole points in Pop(*t*), not just the local part of them. As is known, least squares approach is a simple and effective method for linear curve fitting and nonlinear curve fitting, such as polynomial or exponential curve fitting. Then we consider whether this technique could be made use of to describe expression (2).

For the sake of simplicity, it could be assumed that the centroid of *ξ* is a manifold *ψ* in formula (2), and *ζ* is uniformly distributed on *ψ*. *ψ* is a (*m* − 1)-dimensional hyperrectangle. Particularly, in the case of two objectives, *ψ* is a curve segment in *R* ^2^.

A line in 3-dimensional space can be expressed as

(3) $$\begin{array}{c} x=az+c,\;\;y=bz+d, \end{array}$$

where *a*, *b*, *c*, and *d* are the coefficients of the expression.

**Figure 2:**
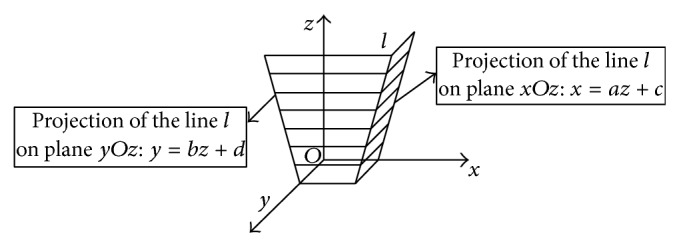
Illustration of the geometric meaning of expression (3).

The geometric meaning of expression (3) is that a 3-dimensional line *l* can be seen as the intersecting line of two planes *m*_1_: *x* = *az* + *c* and *m*_2_: *y* = *bz* + *d*.  Figure 2 illustrates this meaning.

The expression *x* = *az* + *c* can be seen as the projection of the line *l* in *xOz* plane, and *y* = *bz* + *d* is the one in the *yOz* plane.

As a 3-dimensional line can be expressed by the intersecting of 2 planes, then a *n*-dimensional line can be expressed as the intersecting of (*n* − 1) planes as

(4) x2=a1+b1x1,x3=a2+b2x1,⋮xn=an−1+bn−1x1.

Expression *x*_*i*_ = *a*_*i*−1_ + *b*_*i*−1_ *x*_1_ can be regarded as the projection of the *n*-dimensional line in *x*_*i*_ *Ox*_1_ plane.

By expression (4), we further conclude that a *n*-dimensional curve can be regarded as the intersecting of (*n* − 1) surface, and expression (5) shows this idea:

(5) x2=a1,0+a1,1x1+a1,2x12+⋯+a1,px1p,x3=a2,0+a2,1x1+a2,2x12+⋯+a2,px1p,⋮xn=an−1,0+an−1,1x1+an−1,2x12+⋯+an−1,px1p.

Expression *x*_*j*_ = *a*_*j*−1,0_ + *a*_*j*−1,1_ *x*_1_ + *a*_*j*−1,2_ *x*_1_^2^ + ⋯+*a*_*j*−1,*p*_ *x*_1_^*p*^ can be regarded as the approximate projection of the *n*-dimensional curve in *x*_*j*_ *Ox*_1_ surface. Each expression is a *p*-order polynomial. (*x*_1_,…, *x*_*n*_) is a point on the *n*-dimensional curve. Then the thing that we need to do is to find out all the coefficients *a*_*i*,*j*_, which could make the curve fit the population in the decision space well, and here we used least squares approach method to help us find out the best coefficients.

**Figure 3:**
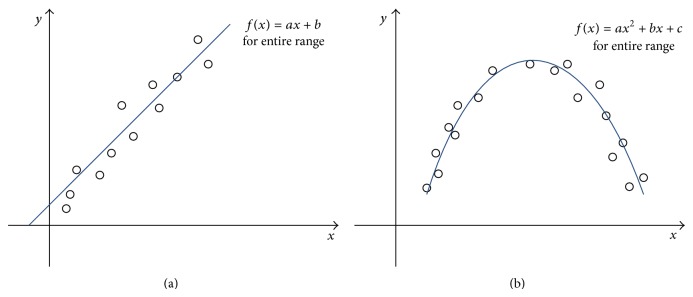
Illustration of the least squares approach (a) linear (b) nonlinear.

Least squares approach is mainly used to fit the curve, that is to say, to capture the trend of the data by assigning a single function across the entire range.  Figure 3 shows the idea.

In Figure 3, Figure 3(a) looks linear in trend, so we can fit the curve by choosing a general form of the straight line *f*(*x*) = *ax* + *b*, and then the goal is to identify the coefficients *a* and *b* such that *f*(*x*) fits the date well, the method to identify the two coefficients is called as linear regression.  Figure 3(b) looks nonlinear, we use higher polynomial *f*(*x*) = *ax* ^2^ + *bx* + *c*, and the goal is to find out the coefficients *a*, *b*, and *c* such that *f*(*x*) fits the date well. It is called as nonlinear regression compared with linear regression. In fact, there are a lot of functions with different shapes that depend on the coefficients. The methods to find out the best coefficients are just called as regression analysis (RA).

Consider the general form for a polynomial with order *j*:

(6) $$\begin{array}{c} f\left(x\right)=a_{0}+a_{1}x+a_{2}x^{2}+\cdots +a_{j}x^{j}=\overset{j}{\underset{k=0}{\sum }}a_{k}x^{k}. \end{array}$$

**Figure 4:**
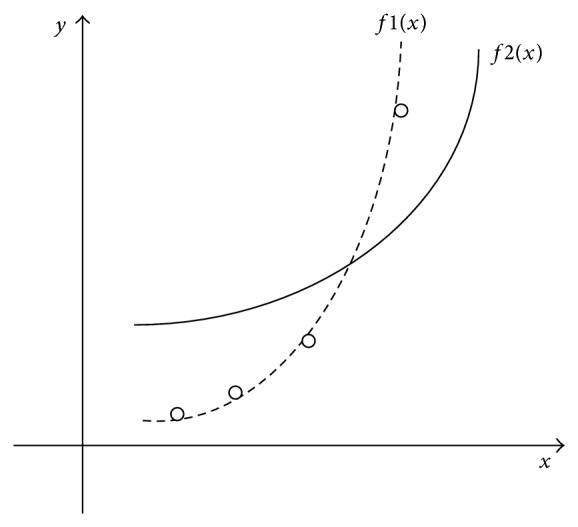
Four data points and two different curves.

How can we choose the coefficients that best fit the curve to the data? The idea of least squares approach is to find a curve that gives minimum error between data *y* and the fitting curve *f*(*x*). As is shown in Figure 4, we can firstly add up the length of all the solid and dashed vertical lines and then pick curve with minimum total error. The general expression for any error using the least squares approach is

(7) err⁡=∑i=1ndi2=y1−fx12+y2−fx22 +⋯+yn−fxn2.

For expression (7), we want to minimize the error err. Replace *f*(*x*) in expression (7) with the expression (6), and then we have

(8) $$\begin{array}{c} \mathrm{err}=\overset{n}{\underset{i=1}{\sum }}{\left(y_{i}-\overset{j}{\underset{k=0}{\sum }}a_{k}x_{i}^{k}\right)}^{2}, \end{array}$$

where *n* is the number of data points given, *i* is the current data points being summed, and *j* is the polynomial order. To find the best line means to minimize the square of the distance error between line and data points. Find the set of coefficients *a*_0_, *a*_1_,…,*a*_*j*_, that is to say, to minimize expression (8).

In Figure 4, there are four data points and two fitting curves *f*1(*x*) and *f*2(*x*). Obviously, *f*1(*x*) is better than *f*2(*x*) because there is smaller error between the four points and the fitting curve *f*1(*x*).

To minimize expression (8), take the derivative with respect to each coefficient *a*_*k*_ for *k* = 0,1,…, *j*, and set each to zero:

(9) ∂err⁡∂a0=−2∑i=1nyi−∑k=0iakxk=0,∂err⁡∂a1=−2∑i=1nyi−∑k=0iakxkx=0,⋮∂err⁡∂aj=−2∑i=1nyi−∑k=0iakxkxj=0.

Rewrite these *j* + 1 equations, and put into matrix form:

(10) n∑xi∑xi2⋯∑xij∑xi∑xi2∑xi3⋯∑xij+1⋮∑xij∑xij+1∑xij+2⋯∑xij+ja0a1⋮aj =∑yi∑xiyi⋮∑xijyi.

The coefficients *a*_*k*_ for *k* = 0,1,…, *j* can be solved by matrix computation.

With the above work, we can describe the 1-dimensional manifold *ψ* as

(11) ψ=x=x1,…,xn∈Rn ∣ ∑j=0pkkkkkxi=∑j=0pai−1,jx1j,  a1−0.25b1−a1kkk11k11k∑j=0p≤x1≤b1+0.25b1−a1,  i=2,…,n,

where *p* is the polynomial order and *a*_1_ and *b*_1_ are the minimum and maximum values on *x*_1_:

(12) $$\begin{array}{c} a_{1}=\underset{1\leq j\leq N}{\min}x_{1}^{j},\;\;b_{1}=\underset{1\leq j\leq N}{\max}x_{1}^{j}. \end{array}$$

**Figure 5:**
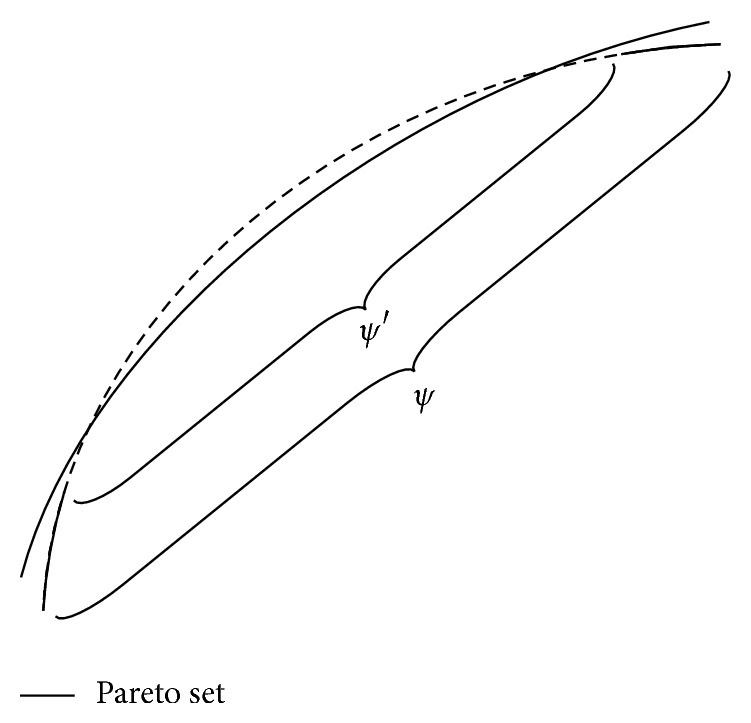
Illustration of extension.

In order to approximate the PS better, *ψ* is extended by 50% along *x*_1_.  Figure 5 shows this idea. In Figure 5, *ψ*′ could not approximate the PS very well, but its extension *ψ* can provide a better approximation.

When we find out the coefficients *a*_*i*,*j*_  (*i* = 1,…, *n* − 1,  *j* = 0,…, *p*) based on least square approach above, we could get *ζ* in expression (2). *ζ* is generated over *ψ* uniformly and randomly.

**Figure 6:**
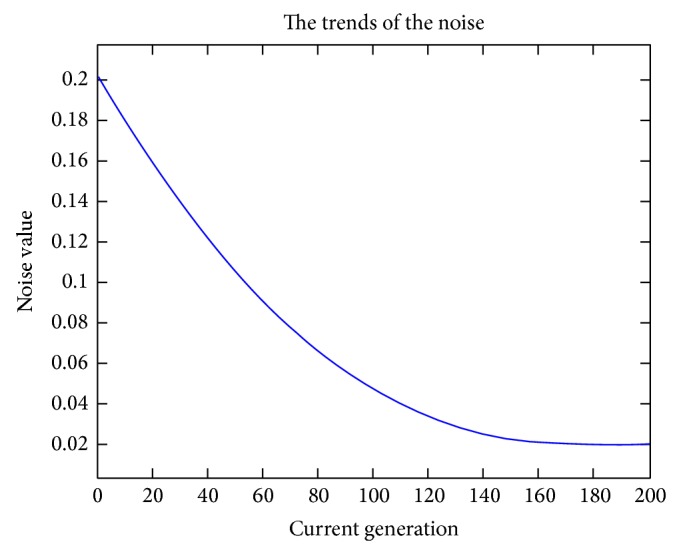
The trends of the noise.

In expression (2), *ε* is a *n*-dimensional zero-mean noise vector, and it is designed as the following description:

(13) $$\begin{array}{c} \varepsilon =\left(\varepsilon_{1},\varepsilon_{2},\ldots ,\varepsilon_{n}\right), \end{array}$$

where *ε*_*i*_ is a random number between (−noise, noise). The noise is changed from big to small as the generation goes on because big noise can accelerate the convergence of the population in the early generation and small noise can maintain the accuracy of the population in the end. Expression (14) shows the implementation:

(14) $$\begin{array}{c} \text{noise}=F_{0}*10^{e^{\left(1-\text{maxGen}/\left(\text{maxGen}+1-\text{curGen}\right)\right)}}, \end{array}$$

where maxGen is the max generation of the algorithm and is set to be 200 and curGen is the current generation. *F*_0_ is set to be 0.2 when the algorithm begins. Then the noise is changed from 0.2 to 0.02. The trends of the noise can be seen in Figure 6. As is shown in Figure 6, the noise decreases as the generation increases, and it will be stable after the 160th generation.

### 3.4. Reproducing

It is desirable that final solutions are uniformly distributed on the PS. Therefore, in order to maintain the diversity of the solution, in this paper, the new solution is generated uniformly and randomly as follows.

**Step 1 .**

Generate a point *x*′ from *ψ* uniformly and randomly.

**Step 2 .**

Generate a noise vector *ε*′ in expression (13).

**Step 3 .**

Return *x* = *x*′ + *ε*′.

In Step 3 of the algorithm framework of MMEA-RA, *N* new solutions can be produced by repeating above all steps *N* times.

### 3.5. Selecting

The selection procedure used in this paper is the same in the procedure used in [5], which is based on the nondominated sorting of NSGA-II [15]. The selection procedure is called as NDS-selection.

The main idea of NDS-selection is to divide *Q* ∪ Pop(*t*) into different fronts *F*_1_, *F*_2_,…, *F*_*l*_ such that the *j*th front *F*_*j*_ contains all the nondominated solutions in {*Q* ∪ Pop(*t*)}∖(⋃_*i*=1_^*j*−1^ *F*_*i*_). Therefore, there is no solution in {*Q* ∪ Pop(*t*)}∖(⋃_*i*=1_^*j*−1^ *F*_*i*_) that could dominate a solution in *F*_*j*_. Roughly speaking, *F*_1_ is the best nondominated front in *Q* ∪ Pop(*t*), and *F*_2_ is the second best nondominated front, and so on. The detailed procedure of NDS-selection can be found in [5].

## 4. Test Case

### 4.1. Performance Metric

In this paper, the performance metric used to evaluate the solutions is the convergence metric *γ*, which is also the common performance metric in multiobjective optimization algorithm [16].

The metric *γ* measures that the solutions will be convergent to a known set of Pareto-optimal solutions. We find a set of 500 uniformly solutions from the true Pareto-optimal front in the objective space. And then to compute the minimum Euclidean distance of each solution from chosen solutions on the Pareto-optimal front. The average of theses distances is used as the metric *γ*.

### 4.2. General Experimental Setting

There are three algorithms employed to solve the test instance for a comparison. These three algorithms are RM-MEDA, NSGA-II, and MMEA-RA, while MMEA-RA is the new algorithm proposed in this paper.

The three algorithms are implemented by C++. The machine used in the test is Core 2 Duo (2.4 GHz, 2.00 GB RAM). The experiment setting is as follows.

 The number of new trial solutions generated at each generation is set to be 100 for all tests.
 The number of decision variables is set to be 30 for all tests.
 Parameter setting in RM-MEDA: the number of cluster *K* is set to be 5 in local PCA algorithm.
 Parameter setting in MMEA-RA: the order is set to be 2.

We run each algorithm independently 10 times for the test instance. The algorithms stop after a given number of generations. The maximal number of generations in three algorithm is 1000.

Table 1 gives the test instance [5]. In the test instance, the feasible decision space is a hyperrectangle. There are nonlinear variable linkages in the test case. Furthermore, the test instance has many local Pareto fronts since its *g*(*x*) has many locally minimal points. It also has some characteristics such as concave PF, nonlinear variable linkage, and multimodal with Griewank function.

If an element of solution *x*, sampled from MMEA-RA or RM-MEDA, is out of the boundary, we simply reset its value to a randomly selected value inside the boundary.

### 4.3. Performance Analysis

**Figure 7:**
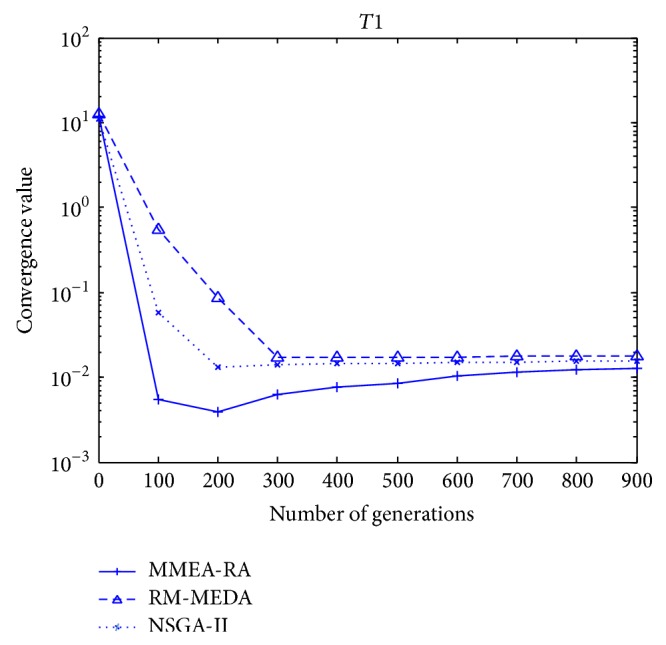
The evolution of the average *γ*-metric of the nondominated solutions in three algorithms for *T*1.

The evolution of the average *γ*-metric of the nondominated solutions for the test case is shown in Figure 7. It should be noted that the solutions of all three algorithms are stable when the iteration generation is more than 300. After the solutions are stable, the convergence values of the three algorithms are small than 0.1. Because we adopt the average of the minimum Euclidean distance of each solution from chosen solutions as the metric *γ*, the smaller the convergence values, the better the convergence metric *γ*. As is shown by Figure 7, among the three algorithms, MEMA-RA has best convergence performance and NSGA-II and RM-MEDA follow.

**Figure 8:**
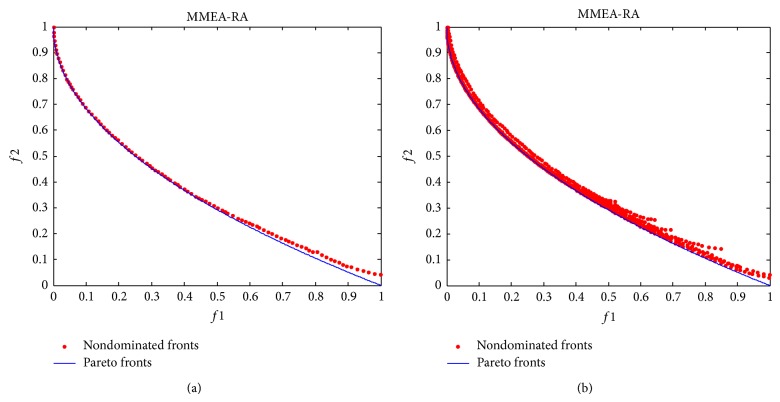
The final nondominated solutions and fronts found by MMEA-RA. (a) The result with the lowest *γ*-metric and (b) all the 10 fronts in 10 runs.

Figure 8 shows the final nondominated solutions and fronts obtained by MMEA-RA on the test case.  Figure 8(a) is the result with the lowest *γ*-metric obtained in 10 runs while Figure 8(b) is all the 10 fronts in 10 runs. It can be seen that the nondominated fronts with the lowest *γ*-metric are very close to the Pareto front, especially when *f*1 tends to 0 and *f*2 tends to 1. It can also be noted that the nondominated solutions in every run have some small fluctuations around the Pareto front.

**Figure 9:**
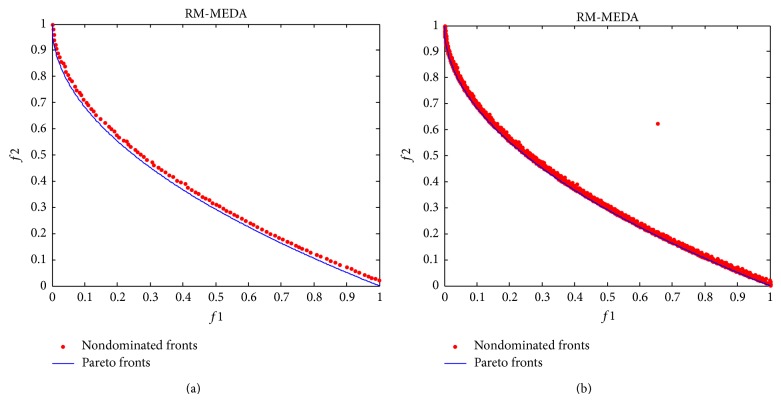
The final nondominated solutions and fronts found by RE-MEDA. (a) The result with the lowest *γ*-metric and (b) all the 10 fronts in 10 runs.

The final nondominated solutions and fronts obtained by RM-MEDA on the test case are shown in Figure 9. Similarly, Figure 9(a) is the result with the lowest *γ*-metric obtained in 10 runs while Figure 9(b) gives all the 10 fronts in 10 runs. Similar to Figure 8, the nondominated solution(s) in Figures 9(a) and 9(b) are marked with red. The Pareto fronts are marked with blue. The Pareto fronts are given in Figures 9(a) and 9(b) only for comparing the quality of the nondominated solutions. It can be seen that the nondominated front with the lowest *γ*-metric is very consistent with the Pareto front although there are some differences between them. In particular, it should be noted that all results in 10 runs from RM-MEDA match the Pareto front better than MMEA-RA. But it also should be noted that there is an isolated point in the nondominated solutions for all 10 runs in Figure 9(b), maybe because RM-MEDA falls into a local minimum and could not jump out.

**Figure 10:**
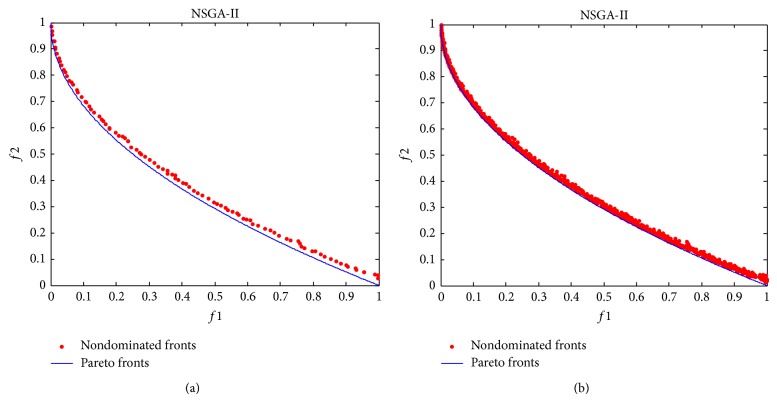
The final nondominated solutions and fronts found by NSGA-II. (a) The result with the lowest *γ*-metric and (b) all the 10 fronts in 10 runs.

The final nondominated solutions and fronts obtained by NSGA-II on the test case are shown in Figure 10. Again, Figure 10(a) means the result with the lowest *γ*-metric obtained in 10 runs and Figure 10(b) means all 10 fronts in 10 runs. As is shown in Figure 10(a), the nondominated front with the lowest *γ*-metric is close to the Pareto front but different to the result obtained by MMEA-RA. The nondominated front with the lowest *γ*-metric in NSGA-II does not tend to the Pareto front very close. It does also not match the Pareto front as good as the result obtained by RE-MEDA. Similarly, the nondominated solutions in every run have some small fluctuations around the Pareto front.

The running time of the three algorithms are given in Table 2. From the point of the running time, as is shown in Table 2, among the three algorithms, NSGA-II is the best, then MMEA-RA follows, and RM-MEDA is the worst. This result is consistent with the main idea of the three algorithms. In RM-MEDA, local principal component analysis (PCA) is used to construct the model, and it needs extra CPU time for running local PCA at each generation. In MMEA-RA, the least squares method is used to construct the model, and it is easy to run the least squares by matrix computation. MMEA-RA is slower than NSGA-II because the selection in MMEA-RA is based on NSGA-II.

Obviously, it can be seen that the nondominated front with the lowest *γ*-metric obtained by MMEA-RA is the closest to the Pareto front in the three algorithms, which shows MMEA-RA is suitable to solve the problem with some characteristics such as concave PF, nonlinear variable linkage, and multimodal with Griewank function. In contrast, the results in 10 runs from RM-MEDA mostly match the Pareto front, which shows the performance of RM-MEDA is good in common.

## 5. Conclusion

In this paper, a model-based multiobjective evolutionary algorithm based on regression analysis (MMEA-RA) is put forward to solve continuous multiobjective optimization problems with variable linkages. MMEA-RA models a promising area whose centroid is a complete and continuous curve described by expression (8). Because of this feature, MMEA-RA does not need to cluster the population. The least squares approach is simple yet enough to describe the nonlinear principal curve using the polynomial model.

The less CPU time of MMEA-RA does not come without a price. MMEA-RA samples points uniformly around the PS in the decision variable space, and the centroid of the model is not piecewise but complete curve. This makes it very difficult for MMEA-RA to approximate the whole PF. The experimental results also reveal that MMEA-RA may fail in test instances with many local Pareto fronts.

The future research topics along this line should include the following points:

1. designing an accurate model to describe the decision space: as the case of 3 objectives, the PS is a surface, so expression (8) cannot solve the problems with 3 objectives right now;
2. combining MMEA-RA with traditional genetic algorithms using operators such as crossover and mutation for accelerating the convergence of the algorithm;
3. improving the method to calculate random noise value to make the final population more convergent;
4. considering the distribution of the solutions in the objective space when sampling solutions from the models to improve the performance of MMEA-RA on the instance;
5. incorporating effective global search techniques for scalar optimization into MMEA-RA in order to improve its ability for global search.

## Figures and Tables

**Table 1:** Test instance.

Test case	Variables	Objectives
*T*1	[0,1]^*n*^ × [0,10]^*n*−1^	*f*_1_(*x*) = *x*_1_ $f_{2}\left(x\right)=g\left(x\right)\left[1-\sqrt{\frac{f_{1}\left(x\right)}{g\left(x\right)}}\right]$ $g\left(x\right)=\frac{1}{4000}\overset{n}{\underset{i=2}{\sum }}{\left(x_{i}^{2}-x_{1}\right)}^{2}-\prod_{i=2}^{n}\cos\left(\frac{x_{i}^{2}-x_{1}}{\sqrt{i-1}}\right)+2$

**Table 2:** The comparison of the running time (unit: ms).

	NSGA-II	RM-MEDA	MEMA-RA
The running time	79.368	127.543	92.771
